# Prognostic Impact of Preoperative Renal Insufficiency on Metastasis-Free Survival after Radical Cystectomy

**DOI:** 10.7150/jca.61847

**Published:** 2021-10-28

**Authors:** Jungyo Suh, Hyeong Dong Yuk, Chang Wook Jeong, Cheol Kwak, Hyeon Hoe Kim, Ja Hyeon Ku

**Affiliations:** 1 Department of Urology, Asian Medical Centre, Seoul, South Korea.; 2 Department of Urology, Seoul National University Hospital, Seoul, South Korea.; 3 Department of Urology, Seoul National University College of Medicine, Seoul, South Korea.

**Keywords:** bladder cancer, metastasis, radical cystectomy, renal insufficiency, survival, urothelial carcinoma

## Abstract

Several studies founded that preoperative renal insufficiency is associated with a higher risk of upper tract urothelial carcinoma recurrence and mortality than normal renal function patients. However, previous studies were all retrospective; no study focused on urothelial carcinoma in the bladder and metastasis-free survival (MFS). Herein, we examined the prognostic impact of preoperative renal insufficiency on the oncologic outcomes of patients with urothelial carcinoma in the bladder after radical cystectomy. We used data from 262 patients prospectively collected from a radical cystectomy cohort between March 2016 and February 2021. The patients were divided into those with a preoperative glomerular filtration rate (GFR) of <60 mL/min/1.73 m^2^ (renal insufficiency; *n*=66) and those with a GFR ≥60 mL/min/1.73 m^2^ (control; *n*=196). We investigated MFS, cancer-specific survival (CSS), and overall survival (OS). Kaplan-Meier curves and Cox proportional hazard regression were used to estimate the prognostic impact of renal insufficiency. The mean MFS was significantly shorter in the renal insufficiency group than in the control group (36.58±3.09 months vs. 47.37±1.87 months); however, OS and CSS were not significantly different. T stage ≥3 (hazard ratio [*HR*]: 2.79), lymph node positivity (*HR*: 2.261), and renal insufficiency (*HR*: 2.04) were significant independent predictors of MFS. Preoperative renal insufficiency was an independent prognostic factor for worse MFS. Well-designed randomized clinical trials and translational studies are needed to clarify the mechanism of relationship between preoperative renal insufficiency and MFS.

## Introduction

Urothelial carcinoma of the bladder (UCB) is the 10^th^ most common cancer worldwide and the 13^th^ leading cause of cancer-related deaths 1. Radical cystectomy (RC) is recommended for muscle-invasive bladder cancer (MIBC) and certain aggressive phenotypes of non-MIBC (NMIBC). However, up to 70% of patients experience local or distant recurrence within two years after complete removal of the primary cancer 2. Distant metastasis decreases life expectancy and requires systemic therapy. Therefore, identifying patients at higher risk of distant metastasis is important, especially among those who require RC. Preoperative renal insufficiency is associated with all-cause mortality in several malignancies 3. A recent meta-analysis found that preoperative renal insufficiency is associated with a higher risk of upper tract urothelial carcinoma (UC) recurrence and mortality 4. Two previous studies reported the effect of preoperative renal insufficiency on oncological outcomes in patients with bladder UC and upper urinary tract UC (UTUC) 5,6. However, these previous studies were based on retrospective data from the 1990s to 2010s and may have been highly vulnerable to recall or sampling biases. Further, the clinical impact of renal insufficiency on metastasis-free survival (MFS) in cases of UCB treated with RC remains unclear.

In this study, we investigated the prognostic impact of preoperative renal insufficiency on MFS in a nonrandomized prospective cohort of patients with UCB. The secondary endpoints were overall survival (OS) and cancer-specific survival (CSS) in patients with preoperative renal insufficiency.

## Materials and Methods

### Ethics Approval and Informed Consent

This study was approved by the Seoul National University Hospital (SNUH) Institutional Review Board (IRB). Informed consent for academic use of the clinical data was obtained from each participant at the time of enrolment in the prospective registry 7. For this ad-hoc study, the SNUH IRB approved the academic use of registry data focused on renal insufficiency and oncological outcomes after RC. All the study processes were performed in accordance with relevant guidelines and regulations.

### Patient selection and cohort follow-up protocols

**Figure 1:**
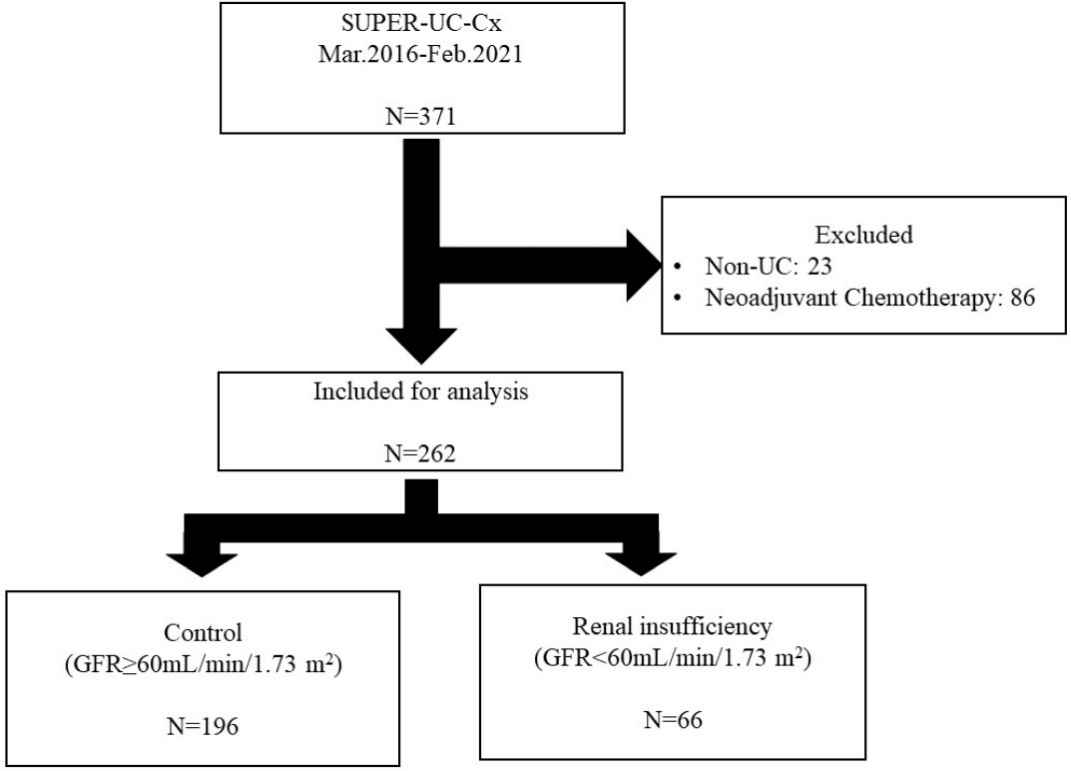
** Flow chart of inclusion and exclusion criteria and patient grouping based on pre-operative estimated glomerular filtration rate.** SUPER-UC-Cx: Seoul National University Hospital Prospectively Enrolled Registry for Urothelial Carcinoma treated by Radical Cystectomy; UC: urothelial carcinoma; GFR: glomerular filtration rate.

For this analysis, we used data from the SNUH Prospectively Enrolled Registry for Urothelial Carcinoma treated with RC, a sub-cohort of the multidisciplinary genitourinary cancer registry of a high-volume tertiary center 7. Patients who underwent RC between March 2016 and February 2021 at SNUH were evaluated. The inclusion criteria were as follows: patients older than 18 years and who underwent RC due to histologically confirmed MIBC or Bacille Calmette Guérin (BCG)-refractory NMIBC. Patients with non-UC on final pathological examination and those who underwent neoadjuvant chemotherapy were excluded (Figure 1). All patients underwent RC with standard pelvic lymph node dissection.

The preoperative glomerular filtration rate (GFR) was calculated using the Modification of Diet in Renal Disease equation (GFR=186.3 × serum creatinine level (mg/dL)^-1.154^ × age^-0.203^ × 0.742 (if female)) 8. Patients were divided into two groups based on the preoperative GFR. Patients with a calculated GFR of <60 mL/min/1.73 m^2^ were assigned to the renal insufficiency group, and those with a calculated GFR of ≥60 mL/min/1.73 m^2^ were assigned to the control group. All the preoperative laboratory tests were performed within one month before RC. If the results of any laboratory tests were abnormal, we repeated those laboratory tests at the time of admission. Patient selection for RC followed current guidelines and recommendations, i.e., clinical T stage ≥2 or treatment-refractory cT1 9. High-risk patients, such as those with pathologic stage T3 or ≥N1, were treated with cisplatin-based adjuvant chemotherapy. Metastasis was assessed using computed tomography every six months for three years after RC and annually thereafter. If the patient had any unusual symptoms, we performed additional laboratory and imaging tests, including positron emission tomography.

### Statistical analysis

The primary endpoint was MFS, while the secondary endpoints were OS and CSS within the follow-up period. Continuous variables were described as means (interquartile ranges), and categorical variables were described as frequencies (percentages). Continuous variables were compared using Student's t-test, and categorical variables were compared using the chi-square test or Fisher's exact test. Kaplan-Meier curves and log-rank tests were used to compare MFS, OS, and CSS of the two groups. Cox proportional hazard regression analysis was performed for MFS, OS, and CSS using known risk factors: age, pathologic T stage, pathologic N stage, sex, history of UTUC, and renal function. Patients were divided into three age groups (<65 years, 65-75 years, and >75 years). Statistical analyses were performed using Python 3.9.0 based on packages dependent on SciPy 10. Statistical significance was set at *p* <0.05, and all reported *p*-values were two-sided.

## Results

### Patient characteristics

A total of 262 patients who underwent RC at SNUH were included in the analysis. Sixty-six patients were assigned to the renal insufficiency group and 196 patients to the control group (Figure 1). The average GFR of the renal insufficiency group was 40.21±15.43 mL/min/1.73 m^2^, and that of the control group was 88.50±19.42 mL/min/1.73 m^2^. The renal insufficiency group was significantly older than the control group (73.64±10.53 years vs. 69.91±10.05 years, *p*=0.01). Sex, body mass index, pathologic T stage, and pathologic N stage were not significantly different between the two groups. Diabetes mellitus and hypertension were found more frequently in the renal insufficiency group, along with a history of UTUC. Laboratory tests showed that renal insufficiency was correlated with low hemoglobin levels, high potassium levels, and high uric acid levels (Table 1).

### Kaplan-Meier survival analysis

**Figure 2:**
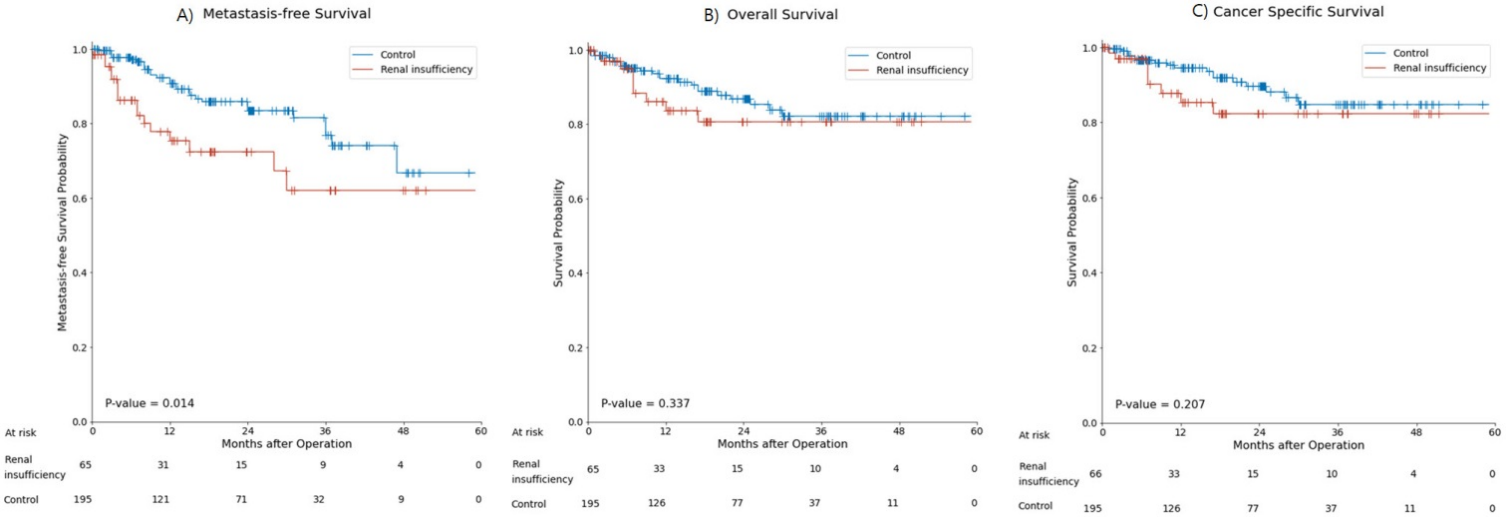
Kaplan-Meier curve for **(A)** metastasis-free survival, **(B)** overall survival, **(C)** and cancer-specific survival in the renal insufficiency and control groups.

During the 18.7±14.4 months of follow-up, 43 patients (16.4%) had metastasis and 31 patients (11.8%) died. Metastasis occurred in 16 patients (24.2%) in the renal insufficiency group and 27 patients (13.7%) in the control group. The mean MFS was significantly shorter in the renal insufficiency group than in the control group (36.58±3.09 months vs. 47.37±1.87 months, *p*=0.01) (Figure 2A). Nine (13.6%) patients died in the renal insufficiency group and 22 (11.2%) died in the control group. The OS and CSS of the renal insufficiency and control groups were not significantly different (Figure 2B, C).

### Univariate and Multivariable Cox proportional hazard regression

The univariate Cox proportional hazard regression analysis indicated that pathologic T stage (≥T3) (hazard ratio [*HR*]: 4.18, 95% confidence interval [*CI*]: 2.28-7.65, *p*<0.01), lymph node metastasis (*HR*: 4.39, 95% *CI*: 2.24-8.60, *p*<0.01) and history of UTUC (*HR*: 2.44, 95% *CI*: 1.08-5.50, *p*=0.03) were significantly associated with MFS. Renal function was also significantly associated with worse MFS (*HR*: 2.13, 95% *CI*: 1.15-3.96, *p*=0.02). In the multivariable analysis, pathologic T stage (*HR*: 2.79, 95% *CI*: 1.35-5.75, *p*<0.01), lymph node metastasis (*HR*: 2.26, 95% *CI*: 1.03-4.96, *p*=0.04), and renal insufficiency (*HR*: 2.04, 95% *CI*: 1.07-3.87, *p*=0.03) remained as independent prognostic factors for MFS (Table 2).

Univariate and Multivariable analyses for OS showed that pathologic T stage (HR: 2.60, 95% CI: 1.12-6.06, *p* = 0.03) and lymph node metastasis (HR: 2.51, 95% CI: 1.05-6.00, *p* = 0.04) were significant independent prognostic factors (Table 3). Univariate and Multivariable analyses for CSS showed that pathologic T stage (HR: 4.94, 95% CI: 2.16-11.32, *p* < 0.01) was the only significant independent prognostic factor (Table 4).

## Discussion

This study investigated the prognostic impact of preoperative renal insufficiency on MFS, OS, and CSS in patients with UCB who underwent RC. In this study, renal insufficiency was not a prognostic factor for OS or CSS. However, renal insufficiency, along with pathologic T stage and lymph node metastasis, were independent prognostic factors for MFS.

Recurrence after RC is relatively common in patients with advanced bladder cancer 11. Although most studies do not distinguish between local recurrence and metastasis, some studies have shown that up to 50% of patients experience distant metastasis after RC 11. A recent study conducted by Park et al. 12 reported that 59.9% of patients with pathologic TanyN1-3M0 disease experienced metastasis within 3 years after RC. The 5-year survival rate of RC varies by initial presentation, from 77% for superficial cancer to 46% for MIBC 13. However, the 5-year survival is notably decreased to less than 15% in patients with metastatic UC 14. Therefore, careful patient stratification and intensive follow-up protocols for high-risk patients are particularly important. In this study, we found that renal insufficiency increases the risk of metastasis. We also identified pathologic T stage and N stage to be significantly associated with metastasis. We suggest that patients with these risk factors should be followed up more carefully for early identification of metastasis.

Renal insufficiency is associated with poor outcomes in UC patients in various ways. A well-established hypothesis suggests that chronic kidney disease (CKD) is a predictor of poor survival in cancer patients because decreased renal function makes the selection or therapeutic dosing of chemotherapeutic agents difficult 15. Most chemotherapeutic agents are nephrotoxic; therefore, patients with advanced CKD experience a vicious cycle of decreasing renal function and decreasing therapeutic effects. Conversely, renal insufficiency can lead to the development of UC and promote the progression of other malignancies. Lin et al. 16 found a higher incidence of oral, colorectal, liver, blood, breast, renal, upper urinary tract, and bladder cancer in patients with end-stage renal disease who were undergoing dialysis than in healthy individuals. Leppert et al. 17 reported that CKD itself increased the risk of UC, although the risk of other malignancies was not significantly increased. A recent meta-analysis showed that postoperative CKD had a poor prognostic impact on UC recurrence and patient survival 4. This study focused on the prognostic impact of preoperative renal insufficiency on MFS after RC in patients with UCB. Renal insufficiency was an independent prognostic factor for MFS (*HR*: 2.04, 95% *CI*: 1.07-3.87, *p*=0.03), although its prognostic impact on OS and CSS was not significant. Owing to a relatively short follow-up period and a limited number of patients, the proportion of deceased patients was only about 10% (31/265 patients) of all patients in this study, which is not enough to determine the prognostic impact of renal insufficiency on OS and CSS in this study. Further studies with a longer follow-up period are warranted; large population-based studies should also be conducted that apply the findings of this study.

The mechanism by which renal insufficiency affects MFS has not been clearly demonstrated and we did not examine it in this study. However, we suggest a potential mechanism of action, where renal insufficiency induces hypoxia and increases toxic agent-mediated cancer cell growth at metastatic sites. Recently, Soave et al. 18 showed that more than 25% of patients with RC have an increased risk of circulating tumor cells (CTCs), which are a potential source of metastasis 19. CTCs in venous blood increase the likelihood of cancer cell migration to solid organs and the progression of metastasis under the proper conditions. In this study, patients with renal insufficiency tended to have lower hemoglobin levels, higher potassium levels, and higher uric acid levels than patients in the control group. Anemia is a well-known complication of CKD 20, and the hypoxia-inducible factor-mediated pathway plays an important role in metastasis 21. Additionally, high uric acid is relatively common in patients with renal impairment 22, and this is a poor prognostic factor for CKD development 22 and cancer progression 23. However, a better-designed study on CKD and metastasis is required to answer these questions.

This study has several limitations. Although we investigated the endpoints in a prospective cohort, we did not perform randomization; therefore, caution should be exercised before applying the results of this study in clinical settings. This study only contained data from a single institution; thus, external validation of the findings is needed. Another important limitation is that data for pre-operative hydronephrosis were not available in this study. Pre-operative hydronephrosis can impact both renal function and prognosis in cases of UCB 24. Moreover, we did not provide a potential mechanism of action by which preoperative renal insufficiency promotes metastasis. Despite these limitations, this is the first study to reveal the effect of preoperative renal insufficiency on MFS after definitive treatment of bladder cancer in a prospective cohort. Well-designed randomized clinical trials and translational studies are needed to clarify the mechanism of action driving this phenomenon.

In conclusion, preoperative renal insufficiency was an independent prognostic factor for shorter MFS. Renal insufficiency was not significantly associated with OS or CSS. Therefore, we suggest careful follow-up in patients with UCB with renal insufficiency before RC. The mechanism underlying the effect of renal insufficiency on MFS is unclear; however, we suggest hypoxia-induced activation of micrometastatic cancer cells. Further investigation is required to clarify this phenomenon.

## Figures and Tables

**Table 1:** Demographics of the renal insufficiency and control groups

	Renal insufficiency	Control	*p*-value
Number of patients	*N* = 66	*N* = 196	
Age, yr.	73.64 (67.8-81.0)	69.91 (62.3-77.0)	0.011
Body mass index, kg/m^2^	23.31 (21.0-25.4)	23.65 (21.0-25.7)	0.482
Diabetes Miletus, *n* (%)	22 (33.3)	38 (19.4)	0.020
Hypertension, *n* (%)	43 (65.2)	91 (46.4)	0.008
History of upper tract urothelial carcinoma, *n* (%)	15 (22.7)	2 (1.0)	<0.001
**Sex, *n* (%)**			0.281
Male	55 (83.3)	151 (77.9)	
Female	11 (16.7)	45 (23.0)	
**Pathologic T stage, *n* (%)**			0.339
pT0	15 (22.7)	30 (15.3)	
pT1	21 (31.8)	73 (37.2)	
pT2	9 (13.6)	39 (19.9)	
pT3-4	21 (31.8)	54 (27.6)	
**Pathologic N stage, *n* (%)**			0.925
pNx and pN0	56 (84.8)	170 (86.7)	
pN1	4 (6.1)	10 (5.1)	
pN2	6 (9.1)	16 (8.1)	
Histologic variants, *n* (%)	10 (19.6)	30 (18.1)	0.805
Lymphovascular invasion, *n* (%)	17 (25.8)	36 (18.5)	0.203
Concomitant carcinoma *in situ*, *n* (%)	20 (30.3)	82 (41.8)	0.096
ADJ chemotherapy, *n* (%)	9 (13.6)	30 (15.3)	0.742
Hemoglobin, g/dL	12.04 (10.8-13.1)	13.10 (11.9-14.3)	<0.001
Potassium, mmol/L	4.56 (4.3-4.8)	4.20 (4.0-4.4)	<0.001
Uric acid, mg/dL	6.11 (5.4-7.1)	5.17 (4.3-6.1)	<0.001

Data are presented as mean (standard deviation; interquartile range) unless otherwise indicated. ADJ: adjuvant.

**Table 2:** Univariate and multivariate Cox proportional hazard regression for metastasis-free survival

Variable	Univariate			Multivariable		
	Hazard ratio	Confidence interval	*p*-value	Hazard ratio	Confidence interval	*p*-value
Age			0.407			
<65 years	Ref					
65-75 years	0.823	0.363-1.867				
>75 years	1.329	0.636-2.777				
Pathologic T stage (≥T3)	4.175	2.277-7.654	<0.001	2.786	1.349-5.754	0.006
Lymph node metastasis	4.390	2.240-8.603	<0.001	2.261	1.031-4.957	0.042
Sex (Female)	1.040	0.524-2.064	0.912			
Renal insufficiency	2.129	1.146-3.956	0.017	2.037	1.074-3.866	0.029
History of UTUC	2.441	1.084-5.500	0.031			

**Table 3:** Univariate and multivariate Cox proportional hazard regression for overall survival

	Univariate			Multivariable		
	Hazard ratio	Confidence interval	*p*-value	Hazard ratio	Confidence interval	*p*-value
**Age**			0.108			0.166
<65 years	Ref					
65-5 years	1.451	0.486-4.332				
>75 years	2.565	0.945-6.965				
Pathologic T stage (≥T3)	4.001	1.957-8.178	<0.001	2.603	1.118-6.061	0.027
Lymph node metastasis	4.366	2.069-9.213	<0.001	2.512	1.052-5.998	0.038
Sex (Female)	0.584	0.224-1.522	0.271			
Renal insufficiency	1.460	0.671-3.175	0.340			0.326
History of UTUC	0.826	0.197-3.464	0.794			

**Table 4:** Univariate and multivariate Cox proportional hazard regression for cancer-specific survival

Variables	Hazard ratio	Confidence interval	*p*-value	Hazard ratio	Confidence interval	*p*-value
**Age**			0.155			0.221
<65 years	Ref					
65-75 years	0.974	0.297-3.193				
>75 years	2.148	0.772-5.976				
Pathologic T stage (≥T3)	5.216	2.301-11.825	0.001	4.941	2.157-11.317	<0.001
Lymph node metastasis	3.866	1.646-9.077	0.002			0.221
Sex (Female)	0.567	0.194-1.653	0.299			
Renal insufficiency	1.708	0.736-3.963	0.213			0.181
History of UTUC	1.035	0.244-4.392	0.963			

## Abbreviations

CSS: cancer-specific survival
GFR: glomerular filtration rate
MFS: metastasis-free survival
MIBC: muscle-invasive bladder cancer
OS: overall survival
RC: Radical cystectomy
UCB: Urothelial carcinoma of the bladder
UTUC: upper urinary tract UC
